# Supplementary material for: Hemodynamic simulation in the aortic arch under anemic, diabetic, and healthy blood flow conditions using computational fluid dynamics
Source: PLoS One. 2026 Mar 11;21(3):e0334297. doi: 10.1371/journal.pone.0334297 (PMC12978756; doi:10.1371/journal.pone.0334297)
Supplement: Attachment — Submitted filename: Response to reviewers.pdf [file pone.0334297.s002.pdf]

For research article

Response to Reviewer Comments

Response to Reviewer 1

We thank Reviewer 1 for the constructive and insightful comments on our manuscript. The suggestions have been carefully considered, and the manuscript has been revised accordingly to improve clarity, methodological rigor, and interpretative precision. Detailed point-by-point responses to each comment are provided below, with corresponding revisions incorporated into the manuscript.

The computational study investigates and compares the hemodynamic effects of anemic, diabetic, and healthy blood conditions within the human aortic arch using a unified simulation framework. The objective is to elucidate how pathological alterations in blood rheology—specifically viscosity and hematocrit—affect key hemodynamic parameters such as velocity, pressure, and wall shear stress (WSS), thereby providing mechanistic insight into cardiovascular risk factors.

Response: *We thank the reviewer for the careful evaluation of our manuscript. The feedback has helped clarify the scope of the study, strengthen the methodological presentation, and refine the interpretation of the results. All comments have been addressed through targeted revisions, as detailed below.*

Comment 1: The use of a constant static pressure at the outlets is a significant simplification. In vivo, the smaller arteries branching from the aorta present complex, dynamic resistance and compliance. This simplification likely affects the accuracy of flow distribution and pressure drops among the branches, which are critical for assessing regional hemodynamics.

Response 1: *We thank the reviewer for this valuable comment. In the revised study, the constant outlet pressure assumption has been replaced with a physiologically realistic three-element Windkessel (RCR) model at each of the four outlets. This improvement enables the outlet pressure to vary dynamically according to the instantaneous flow rate, capturing the resistive and compliant behavior of the downstream vasculature and eliminating the limitations of a fixed boundary. The aortic arch comprises four outlets: the brachiocephalic (O_1), left common carotid (O_2), left subclavian (O_3), and descending aorta (O_4), each assigned its own RCR parameters $(R_{1,i}, R_{2,i}, C_i)$ representing proximal resistance, distal resistance, and compliance [4, 6, 10]. The outlet pressure $P_i(t)$ was related to the instantaneous flow $Q_i(t)$ through the Windkessel formulation $P_i(t) = P_{c,i}(t) + R_{1,i}Q_i(t)$, and discretized using a backward-Euler scheme to ensure numerical stability. Under these pulsatile inlet and RCR outlet conditions, the simulated pressure and flow fields exhibit physiologically realistic wave propagation, damping, and branch-specific flow distribution, addressing the reviewer's concern regarding the oversimplification of outlet boundary conditions. Accordingly, all pressure- and flow-related figures, tables, and associated discussions have been*

updated to reflect the revised RCR outlet boundary conditions.

"The aortic arch model comprises four outlets: the brachiocephalic artery (O_1), left common carotid artery (O_2), left subclavian artery (O_3), and descending aorta (O_4). Each outlet was coupled with a three-element Windkessel (RCR) model to represent downstream vascular resistance and compliance [4, 6, 10]. This model provides a physiologically realistic pressure–flow coupling by accounting for both the resistive and compliant effects of the peripheral vasculature, allowing outlet pressure to evolve dynamically with time rather than remain fixed. Each branch was assigned parameters $(R_{1,i}, R_{2,i}, C_i)$, denoting proximal resistance, distal resistance, and compliance, respectively, and related through the Windkessel formulation $P_i(t) = P_{c,i}(t) + R_{1,i}Q_i(t)$, where $P_{c,i}$ varies with capacitive storage and distal resistance. The governing relation was discretized using a first-order backward-Euler scheme to ensure numerical stability:

$$P_i^{n+1} = \frac{\beta_i P_i^n + Q_i^{n+1}(R_{2,i} + R_{1,i} + R_{1,i}\beta_i) - R_{1,i}\beta_i Q_i^n}{1+\beta_i}, \qquad \beta_i = \frac{R_{2,i}C_i}{\Delta t}. \tag{1}$$

The updated pressure P_i^{n+1} was applied at each time step as the outlet boundary condition, allowing physiological wave reflections and pressure decay throughout the cardiac cycle. The total resistance for each branch was estimated as $R_{\text{tot},i} = \bar{P}_{\text{mean}}/\bar{Q}_{i,\text{mean}}$, with $R_{1,i} = 0.05\,R_{\text{tot},i}$, $R_{2,i} = 0.95\,R_{\text{tot},i}$, and $C_i = \tau/R_{2,i}$, where $\tau \approx 1.0\,\text{s}$ is the diastolic decay constant. The mean flow partitions among the four outlets followed the ratios reported by Reymond *et al.* [10]."

"Pressure distribution in the aortic arch provides critical insights into the flow resistance and hemodynamic forces acting on the arterial walls. Under pulsatile inlet conditions with RCR outlet coupling, the simulated pressure field reflects physiologically realistic wave propagation and damping across the arch. Abnormal pressure gradients are clinically associated with increased afterload, impaired organ perfusion, and progressive cardiovascular disease, particularly in patients with hypertension or systemic vascular stiffening [11]."

Comment 2: The aortic model is relatively smooth and assumes a non-diseased state. Consequently, the findings may not be generalizable to patients with common pre-existing conditions like atherosclerotic plaques, stenosis, or aneurysms, where geometric abnormalities dramatically alter local flow patterns and WSS.

Response 2: *We thank the reviewer for this comment. We acknowledge that the use of a smooth, non-diseased aortic geometry limits direct generalization of the present findings to patients with geometric pathologies such as atherosclerotic plaque, stenosis, or aneurysms, where local flow patterns and wall shear stress can be substantially altered. This limitation has been explicitly noted in the revised manuscript. As clarified in the Conclusion, extension of the present framework to patient-derived and pathological geometries, along with compliant arterial wall modeling, is identified as an important direction for future research.*

"Future research should address these limitations by ~~integrating patient-derived geometries~~ extending the present framework to patient-derived vascular geometries, incor-

porating compliant wall models through FSI, and expanding simulations to pathological cases such as stenosis and aneurysm. The present study is intentionally restricted to a non-diseased aortic geometry to establish a hemodynamic baseline, upon which pathological conditions can be systematically introduced."

Comment 3: The study employs standardized parameters from the literature to define "anemic," "diabetic," and "healthy" blood. While based on real data, this approach treats each condition as a single, uniform state. In clinical reality, these conditions exist on a wide spectrum of severity, leading to a corresponding range of blood viscosities. A more nuanced analysis considering this variability would enhance the clinical relevance of the findings.

Response 3: *We thank the reviewer for this valuable comment. We agree that anemic, diabetic, and healthy blood conditions exist along a continuum of severity in clinical practice, with corresponding variability in rheological properties. In the present study, this variability is addressed by considering multiple representative rheological states and by introducing pressure- and wall shear stress–based risk classification metrics, which provide a comparative assessment of hemodynamic severity across conditions rather than relying on single-point indicators alone. While the present study does not perform a continuous parameter sweep over hematocrit or viscosity, the selected cases are intended as representative anchor points spanning clinically relevant rheological extremes.*

Specifically, the inclusion of minimum, maximum, and average pressure and WSS metrics, together with risk-based categorization, enables a more nuanced interpretation of hemodynamic consequences associated with different blood rheologies under identical geometric and boundary conditions. This approach allows clinically relevant distinctions to be drawn among representative physiological and pathological blood states while maintaining a controlled and interpretable modeling framework.

We acknowledge that a fully continuous severity analysis—such as systematic parameter sweeps over hematocrit or viscosity ranges, or comparison across multiple rheological constitutive models—would further enhance clinical relevance. Such extensions are identified as important directions for future work, building upon the present framework.

"Incorporating alternative rheological models like Herschel–Bulkley or Casson ~~could improve accuracy in diseased states~~ or more comprehensive constitutive formulations such as thixo–elasto–visco–plastic (TEVP) models could improve accuracy in diseased states [7, 8]."

Comment 4: The Carreau-Yasuda model effectively captures the shear-thinning behavior of blood but neglects other non-Newtonian characteristics. Recent advances in hemorheology, as detailed in works by Giannokostas and colleagues (e.g., J. Non-Newton. Fluid Mech., 2023; 2021; Materials, 2020), highlight the importance of properties like viscoelasticity, yield stress (viscoplasticity), and thixotropy. These properties are particularly critical in low-shear and recirculation zones, which are pivotal for understanding phenomena like thrombus formation. Incorporating these advanced models would substantially improve the physiological fidelity of the simulations. Furthermore, to accurately capture the

complex hematological variations that distinguish anemic, diabetic, and healthy blood, a Thixo-Elasto-Visco-Plastic (TEVP) model is necessary. Such a model can uniquely account for the underlying rheological changes driven by variations in hematocrit, as well as the aggregability of red blood cells (RBCs) and platelets.

Response 4: *We thank the reviewer for this insightful comment and for highlighting recent advances in hemorheological modeling. We agree that while the Carreau–Yasuda model effectively captures shear-thinning behavior, it does not represent additional non-Newtonian characteristics such as viscoelasticity, yield stress, or thixotropy, which may become important in low-shear and recirculation regions. More comprehensive constitutive formulations, including thixo–elasto–visco–plastic (TEVP) models as discussed by Giannokostas and co-workers, are well suited to represent these effects and hematocrit-dependent aggregation phenomena.*

In the present study, the Carreau–Yasuda model was selected because it provides a well-established and widely used description of blood rheology using parameter sets that are available and comparable for anemic, diabetic, and healthy blood conditions. Implementation of TEVP-type models would require additional clinically resolved parameters such as yield stress, elastic relaxation times, and thixotropic structure evolution coefficients, which typically necessitate dedicated clinical or rheometric studies and are therefore beyond the scope of the present work. Accordingly, incorporation of TEVP models, together with acquisition of the required clinical parameters and systematic comparison with Carreau–Yasuda based predictions, is identified as a subject for future, dedicated investigations, as now noted in the revised Conclusion.

"Incorporating alternative rheological models like Herschel–Bulkley or Casson ~~could improve accuracy in diseased states~~ or more comprehensive constitutive formulations such as thixo–elasto–visco–plastic (TEVP) models could improve accuracy in diseased states [7, 8]."

Comment 5: The authors note that experimental validation was beyond the study's scope; however, this remains a critical step for establishing model credibility. Corroborating the results with experimental data (e.g., via particle image velocimetry) or clinical imaging (e.g., 4D Flow MRI) is essential to transition the predictions from theoretical to validated findings.

Response 5: *We thank the reviewer for this important comment. We fully agree that experimental or clinical validation is a critical step for establishing the predictive credibility and clinical relevance of computational hemodynamic models. In the present study, validation using particle image velocimetry (PIV) or clinical imaging modalities such as 4D Flow MRI was beyond the scope, as it would require dedicated experimental setups or access to patient-specific imaging data and associated ethical approvals.*

The primary objective of this work was to develop and analyze a controlled computational framework to examine the hemodynamic consequences of different blood rheological states under physiologically realistic boundary conditions. Nevertheless, corroboration of the numerical predictions with experimental or in vivo measurements remains essential for clinical translation. Accordingly, experimental and imaging-based validation is identified as an important direction for future work, as now explicitly stated in the revised manuscript.

”Experimental validation was beyond the scope of this work but remains essential to confirm key predictions but remains essential to confirm key predictions through experimental (e.g., particle image velocimetry) or clinical imaging (e.g., 4D Flow MRI) studies, particularly regarding shear stress thresholds and risk classification.”

Comment 6: The risk classification tables (Figures 9b and 11b) offer an effective visualization of the hemodynamic findings. It is important, however, to explicitly frame them as illustrative conceptual tools rather than validated clinical instruments. Clinical risk is a multifactorial continuum influenced by genetics, systemic inflammation, and lipid profiles, factors beyond the scope of this purely hemodynamic assessment.

Response 6: *We thank the reviewer for this helpful comment and for the positive assessment of the risk classification tables. We agree that the pressure- and wall shear stress–based classifications presented in Figures 10b and 12b should be interpreted as illustrative, conceptual tools rather than validated clinical risk instruments.*

In response, we have revised the figure captions and associated discussion to explicitly frame these classifications as relative, hemodynamic indicators intended for comparative and mechanistic interpretation only. The terminology has been carefully adjusted to emphasize that the color-coded categories represent illustrative hemodynamic severity rather than clinical risk prediction, and that the analysis does not account for other multifactorial contributors to clinical risk such as genetics, systemic inflammation, or lipid profiles. These clarifications ensure that Figures 10b and 12b are interpreted strictly within the scope of a purely hemodynamic assessment, consistent with the reviewer's recommendation.

”Figure 10b provides a categorical risk assessment categorical hemodynamic risk assessment intended as an illustrative tool based on minimum, maximum, and average wall pressure metrics. The classification logic is physiologically informed and intended to capture deviations from optimal hemodynamic conditions in a relative, comparative sense rather than as a clinically validated predictor. In pulsatile aortic blood flow, the minimum and maximum luminal pressures could be interpreted as approximations of diastolic and systolic pressures, respectively. Although CFD simulations typically do not model arterial wall compliance unless coupled with structural mechanics, these pressure extrema still offer useful proxies for clinical pressure phases hemodynamic proxies for comparative analysis when performing comparative risk analysis. The classification matrix emphasizes key clinical contrasts hemodynamic contrasts across blood rheological states. The diabetic profile is flagged for high-pressure risk elevated hemodynamic pressure severity, due to its elevated maximum wall pressure, consistent with increased vascular resistance and strain on the arterial wall. It also shows a borderline response in minimum and average pressure, suggesting incomplete normalization even during diastolic phases [11]. The anemic condition is marked by low-pressure risk reduced pressure severity, particularly in the minimum metric, which reflects reduced circulatory force and potential for inadequate capillary perfusion. Both healthy cases, particularly Healthy 2 fall predominantly in the no-risk zone low-severity zone, reinforcing the interpretation that these conditions exhibit stable, well-regulated hemodynamics supportive of systemic

vascular health."

"Figure 12(a) quantifies these observations. The diabetic case yields the highest maximum WSS (6.01 Pa), exceeding commonly reported physiological ranges, while the anemic case shows the lowest average (0.041 Pa) and minimum (0.005 Pa) WSS. Healthy cases remain mostly within the accepted physiological range of 0.4 to 1.5 Pa [9]. Panel (b) translates these metrics into a color-coded ~~risk map~~ illustrative hemodynamic severity map: the diabetic case shows ~~high-WSS risk~~ elevated WSS severity, while the anemic case approaches the low-WSS threshold (yellow). The healthy cases are within the ~~safe zone~~ low-severity zone across all three metrics. These results confirm that blood rheology modulates the local hemodynamic environment, with ~~clinical implications for cardiovascular risk~~ important hemodynamic implications relevant to vascular health. Anemia leads to sub-threshold WSS that may impair mechanosensitive endothelial responses, whereas diabetes imposes excessive WSS variations that can cause mechanical endothelial stress. These findings reinforce the role of WSS as a ~~predictive biomarker for vascular dysfunction~~ hemodynamic indicator associated with vascular dysfunction and ~~support its clinical application in disease risk stratification~~ support its use as a comparative hemodynamic metric in mechanistic studies [13, 14]. From a translational perspective, ~~identifying regions of abnormal WSS can inform patient-specific risk~~ identifying regions of abnormal WSS can inform relative susceptibility for intimal thickening, aneurysmal remodeling, or thrombus initiation, particularly in high-risk groups such as diabetics and anemic individuals [2, 5]."

—

Before closing, we would like to express our sincere thanks for your thoughtful advice and careful review, which have greatly helped us improve the quality and clarity of our paper. We hope our revisions and explanations meet your expectations. Thank you once again.

Response to Reviewer 2

We sincerely thank Reviewer 2 for the constructive and thorough evaluation of our work. In revising the manuscript, we carefully addressed all comments by improving clarity and consistency, refining the physical interpretation of the results, enhancing figure descriptions and readability, and strengthening the linkage between hemorheology and hemodynamics under pulsatile flow. We greatly appreciate the reviewer's feedback, which has helped improve the overall quality of the manuscript.

This paper simulated the flow based on CFD in the human aortic arch for anemic, diabetic, and two healthy subjects using the Carreau–Yasuda model of hemorheology under pulsatile flow. Even though this study combines hemodynamics and hemorheology with quantitative analysis, this requires extensive revision before publication.

Response: *We thank the reviewer for the constructive comments. The manuscript has been extensively revised to improve clarity, consistency, and physical interpretation, including enhanced figure descriptions, the addition of viscosity–shear-rate visualization, and clearer distinction between the roles of hematocrit and viscosity in pulsatile aortic flow.*

Comment 1: Compared with previous published papers such as [1] & [3] in the reference, what is the originality of this paper? The authors should persuade this more rigorously.

Response 1: *We thank the reviewer for raising this important question regarding originality. While prior studies such as Attia [3] and Ahmed [1] have investigated blood rheology using CFD, they addressed different scientific objectives and hemodynamic regimes than the present work. Attia [3] primarily focused on non-Newtonian rheological modeling and viscosity parameterization, with emphasis on constitutive behavior rather than disease-specific hemodynamic consequences in complex arterial domains. Ahmed [1], our prior study, adopted a comparative rheological framework to examine carotid artery flow, a branch-level arterial system where hemodynamics are predominantly governed by local shear-driven mechanisms.*

The originality of the present study lies in its controlled, system-level investigation of pathological blood rheology within the aortic arch, a central arterial conduit characterized by strong curvature-induced secondary flows, inertial effects, and pressure redistribution across multiple outlets. By systematically isolating blood rheology as the sole varying parameter, while employing identical geometry, pulsatile inflow, and physiologically realistic three-element Windkessel (RCR) outlet boundary conditions, this work provides mechanistic insight into how pathological blood properties reshape pressure, velocity, and wall shear stress organization. These system-level effects cannot be inferred from branch-level or single-condition analyses. We have clarified this distinction and the specific contributions of the present study explicitly in the revised Introduction, as suggested by the reviewer.

"In this study, we address this gap by conducting a rheology-aware CFD analysis of blood flow in the aortic arch, comparing anemic, diabetic, and healthy conditions within a consistent simulation framework. ~~A Carreau viscosity model is used to represent shear-dependent behavior~~ A Carreau–Yasuda viscosity model is employed as a unified constitu-

tive description to enable controlled comparison across blood conditions, and a pulsatile inlet condition is applied to capture physiologic flow dynamics. ~~The simulation resolves transient velocity profiles, pressure distributions, and WSS patterns~~ The simulation resolves transient velocity profiles, pressure distributions, and wall shear stress patterns, to assess how hematocrit- and viscosity-driven rheological variations influence mechanical stress and flow organization. ~~Unlike prior work, this study presents a direct comparison of pathological and physiological blood states under identical boundary and geometric conditions~~ By systematically isolating blood rheology as the primary varying factor, and coupling each outlet to a physiologically realistic three-element Windkessel (RCR) model, the present framework enables mechanistic interpretation of pressure–velocity–WSS coupling in a central arterial conduit. ~~allowing for clear interpretation of rheological effects on vascular mechanics~~ This study provides a controlled, system-level assessment of how pathological blood rheology alone reshapes pressure, velocity, and shear organization in the aortic arch, revealing mechanistic effects that are not accessible from branch-level or single-condition analyses."

Comment 2: The authors claimed that this CFD study is a direct comparison of pathological and physiological blood states for rheological interpretation under identical boundary and geometric conditions. However, it's not clear how pathological blood would be reflected in the rheological effects in CFD. If authors meant that Carreau viscosity model with different coefficients according to pathology, are they good enough? Especially under pulsatile flow, red blood cell aggregation is not just a function of shear rate only but also a function of time and radial and axial positions depending on acceleration and hematocrit. Red cell aggregations are also dependent on axial shear rate in addition to radial shear rate under pulsatile flow. How these complicated red cell aggregation phenomena would be reflected in this simple model? Please state this with references.

Response 2: *We thank the reviewer for this important clarification. In the present study, pathological blood states are represented through disease-specific parameterization of the Carreau–Yasuda viscosity model, which provides an effective shear-rate–dependent description of blood rheology. Variations in hematocrit and pathology are reflected through established differences in the model coefficients, allowing controlled comparison of macroscopic hemodynamic quantities under identical geometric and boundary conditions.*

The Carreau–Yasuda model is widely used for large-artery CFD and is known to adequately capture bulk shear-thinning behavior relevant to wall shear stress estimation under pulsatile flow. We acknowledge that time-dependent red blood cell aggregation, shear-history effects, and axial or radial aggregation dynamics are not explicitly resolved by this class of models. Capturing such phenomena would require more advanced constitutive formulations, such as thixo–elasto–visco–plastic (TEVP) models, together with additional clinically resolved parameters, which are beyond the scope of the present study [7, 8].

Accordingly, this work is intended as a system-level, rheology-aware comparison of pathological and physiological blood states using an effective viscosity framework, with the limitations and future extension to TEVP-type models now explicitly acknowledged in the revised manuscript.

"To incorporate the shear-thinning behavior of blood, the dynamic viscosity μ is de-

fined using the Carreau–Yasuda model:

$$\mu_{\text{eff}}(\dot{\gamma}) = \mu_\infty + (\mu_0 - \mu_\infty)\left(1 + (\lambda\dot{\gamma})^2\right)^{\frac{k-1}{2}} \tag{2}$$

where $\dot{\gamma}$ is the local shear rate, μ_0 and μ_∞ are the viscosities at zero and infinite shear rate, respectively, λ is a time constant, and k is the power-law index. This model effectively captures blood's shear-thinning behavior through an effective viscosity formulation, particularly in the near-wall and low-flow regions common in curved arterial segments."

" Additionally, while the Carreau model captures shear-thinning effects, it does not account for pathological viscoplasticity seen in conditions like thrombosis or inflammation. Moreover, shear-rate–dependent viscosity models do not explicitly resolve time-dependent red blood cell aggregation, shear-history effects, or spatial variations in aggregation dynamics that may arise under pulsatile flow. Experimental validation was beyond the scope of this work ~~but remains essential to confirm key predictions~~ but remains essential to confirm key predictions through experimental (e.g., particle image velocimetry) or clinical imaging (e.g., 4D Flow MRI) studies, particularly regarding shear stress thresholds and risk classification."

"Future research should address these limitations by ~~integrating patient-derived geometries~~ extending the present framework to patient-derived vascular geometries, incorporating compliant wall models through FSI, and expanding simulations to pathological cases such as stenosis and aneurysm. The present study is intentionally restricted to a non-diseased aortic geometry to establish a hemodynamic baseline, upon which pathological conditions can be systematically introduced. Incorporating alternative rheological models like Herschel–Bulkley or Casson ~~could improve accuracy in diseased states~~ or more comprehensive constitutive formulations such as thixo–elasto–visco–plastic (TEVP) models could improve accuracy in diseased states [7, 8]."

Comment 3: Discussion needs to be separated from the results, and limitations need to be part of discussion rather than conclusion.

Response 3: *We thank the reviewer for the comment on manuscript organization. The Results and Discussion are intentionally presented in an integrated manner to maintain a clear physical interpretation of the findings, where interpretation is inherently tied to flow features. The discussion is incorporated throughout the results and explicitly synthesized at the end of the section through comparison with prior literature and clinical context. The limitations and future research directions are summarized in the Conclusion to provide a coherent synthesis of scope, constraints, and outlook without interrupting the results-driven narrative. This structure has been retained to preserve clarity and logical flow.*

Comment 4: For governing equations, please clarify whether they are in cylindrical coordinate or cartesian coordinate including helicity eq. (5), For Eqs (3) & (4), n are not the same so different notation is required.

Response 4: *We appreciate the reviewer's insightful comment. The coordinate framework and notation have been clarified for consistency throughout the manuscript. Specifically, all governing*

equations, including the helicity formulation, are now explicitly stated to be expressed in Cartesian coordinates. The wall-shear-stress expression has also been revised to align with this coordinate system. In addition, to avoid symbol repetition, the power-law index in the Carreau–Yasuda model and the cardiac-cycle index in the pulsatile inlet condition now use distinct symbols. The relevant revised texts are provided below:

"The governing equations for mass and momentum conservation are given in Cartesian co-ordinates as follows:

$$\nabla \cdot \mathbf{u} = 0 \tag{3}$$

$$\rho \frac{\partial \mathbf{u}}{\partial t} + \rho(\mathbf{u} \cdot \nabla)\mathbf{u} = -\nabla p + \nabla \cdot \boldsymbol{\tau} \tag{4}$$

where $\mathbf{u}$ is the velocity field, p is pressure, ρ is the density of blood, and $\boldsymbol{\tau} = \mu(\dot{\gamma}) \left(\nabla \mathbf{u} + \nabla \mathbf{u}^T\right)$ is the extra stress tensor with shear-rate dependent viscosity."

"To quantify near-wall hemodynamics, the wall shear stress τ_{wss} is the tangential force per unit area exerted by the fluid on the arterial wall, given by $-\mu_{\text{eff}} \left(\partial u_t / \partial \eta\right)_{\text{wall}}$, where μ_{eff} is the effective viscosity, u_t is the tangential velocity, and η is the local surface-normal direction."

"To incorporate the shear-thinning behavior of blood, the dynamic viscosity μ is defined using the Carreau–Yasuda model:

$$\mu_{\text{eff}}(\dot{\gamma}) = \mu_\infty + (\mu_0 - \mu_\infty) \left(1 + (\lambda \dot{\gamma})^2\right)^{\frac{k-1}{2}} \tag{5}$$

where $\dot{\gamma}$ is the local shear rate, μ_0 and μ_∞ are the viscosities at zero and infinite shear rate, respectively, λ is a time constant, and k is the power-law index."

Comment 5: What kind of medical imaging data was used? More specific description is required in line 130.

Response 5: *We thank the reviewer for this comment. We have revised the Numerical Methodology section to provide a more specific description of both the imaging origin of the geometry and the mesh refinement procedure. The aortic arch geometry is now explicitly described as being derived from publicly available MRI-based anatomical reconstructions, rather than from subject-specific clinical imaging data, and its provenance is clarified through reference to the publicly available CAD source used in this study. In addition, the description of the grid sensitivity analysis has been expanded to specify the range of mesh resolutions tested and the hemodynamic quantities used to assess convergence. These clarifications have been incorporated at the indicated location in the revised manuscript.*

"~~Several meshes were tested by varying the maximum element size while holding all physical and boundary conditions constant.~~ Several unstructured tetrahedral meshes were tested by varying the maximum element size from 4.0 mm to 0.7 mm, while holding all physical models and boundary conditions constant, in order to assess convergence of outlet velocity and wall shear stress predictions."

"The geometry used in this study was obtained from a publicly available CAD model (https://grabcad.com/library/human-aortic-arch-1) and refined to eliminate non-physiological surface irregularities."

"To perform physiologically accurate simulations of blood flow in the aortic arch, the computational domain was constructed from a ~~patient-inspired geometry, originally derived from medical imaging data~~ patient-inspired aortic arch geometry derived from publicly available MRI-based anatomical reconstructions, rather than from subject-specific clinical imaging data and converted into a surface mesh in STL format."

Comment 6: In Fig. 2, (a) is not required, but (b) with more specific mesh and grid should be included in addition to overall mesh map.

Response 6: *We thank the reviewer for the suggestion. Figure 2 has been revised to include enlarged views showing detailed mesh refinement at key regions, in addition to the overall mesh, thereby improving clarity of the grid resolution used in the simulations.*

Comment 7: Table 1 & 2, it not clear why 1mm of grid size and 0.4 s are optimal.

Response 7: *We thank the reviewer for the comment. The selection of a maximum grid size of 1.0 mm and a time step of 0.4 s is based on systematic spatial and temporal sensitivity analyses presented in Tables 1 and 2. As shown in the revised manuscript, multiple unstructured meshes were tested by progressively refining the maximum element size from 4.0 mm to 0.7 mm, while keeping all physical models and boundary conditions fixed. The results demonstrate that variations in outlet velocities and wall shear stress become negligible beyond a mesh size of 1.0 mm, indicating spatial convergence, while further refinement increases computational cost without improving accuracy. Similarly, the time-step sensitivity study shows that key hemodynamic quantities remain unchanged for time steps smaller than 0.4 s under a fixed total simulation duration, confirming temporal convergence. The manuscript has been revised to explicitly clarify these convergence trends and to justify the selected discretization parameters as an optimal balance between numerical accuracy and computational efficiency.*

"To verify that flow predictions were independent of spatial discretization, a grid sensitivity analysis was conducted. This test was crucial to ensure that numerical results, particularly velocity and wall shear stress (WSS), remained consistent as the mesh was refined. ~~Several meshes were tested by varying the maximum element size while holding all physical and boundary conditions constant.~~ Several unstructured tetrahedral meshes were tested by varying the maximum element size from 4.0 mm to 0.7 mm, while holding all physical models and boundary conditions constant, in order to assess convergence of outlet velocity and wall shear stress predictions. As shown in Table 1, changes in key hemodynamic quantities diminished beyond a mesh size of 1.0 mm, with further refinement producing negligible variation, confirming convergence. Accordingly, the selected mesh represents an optimal compromise between numerical accuracy and computational cost, enabling accurate yet efficient simulations."

"To validate the temporal resolution, a time-step sensitivity analysis was carried out. This analysis ensures that the temporal discretization does not distort the time-dependent

solution. Six time steps were tested under constant total simulation duration, and their effects on peak velocity and WSS were evaluated. As shown in Table 2, the variation across step sizes was negligible, confirming that a time step of 0.4 s is sufficient for resolving the pulsatile dynamics while keeping computational effort reasonable."

Comment 8: In addition to Table 3, the graphs are required to figure it out how viscosity would be changed with shear rate for 4 different blood samples.

Response 8: *We thank the reviewer for this suggestion. To improve clarity and provide a direct visualization of the shear-dependent rheological behavior, a new figure (Figure 3) has been added to the manuscript. This figure illustrates the variation of effective blood viscosity with shear rate for all four blood conditions using the Carreau–Yasuda model defined in Eq. (5). The added plot complements Table 3 by clearly demonstrating the shear-thinning behavior of each blood sample and highlighting the differences between anemic, diabetic, and healthy blood profiles. The corresponding description has also been included in the manuscript to explain how these viscosity variations influence the momentum balance, velocity gradients, and wall shear stress distributions.*

"Figure 3 illustrates the variation of effective blood viscosity with shear rate for the four blood conditions computed using the Carreau–Yasuda formulation in Eq. (5). In all cases, viscosity decreases monotonically with increasing shear rate, demonstrating the characteristic shear-thinning behavior of blood. Diabetic blood exhibits the highest viscosity across the entire shear-rate range due to elevated zero-shear viscosity and a delayed transition to the high-shear plateau, whereas anemic blood shows the lowest viscosity with a more rapid reduction as shear rate increases. The two healthy blood cases fall between these extremes, indicating moderate shear-thinning behavior and inter-individual rheological variability. Through Eq. (5), these shear-dependent viscosity variations directly modulate the viscous stress term in the momentum equations, thereby influencing local velocity gradients and wall shear stress distributions, particularly in near-wall and low-shear regions of the aortic arch."

Comment 9: In line 197 & 198, vessel geometry 197 shape overall flow characteristics should be rewritten.

Response 9: *We thank the reviewer for the comment. The text in lines 197–198 has been revised to improve clarity by replacing the ambiguous phrase "vessel geometry shape" with a more precise reference to the arterial shape of the aortic arch and its influence on flow characteristics.*

"Figures 4 and 5 compare velocity streamlines and vector fields, respectively, across four blood conditions: anemic, diabetic, and two healthy cases. These visualizations highlight how blood viscosity, hematocrit level, and ~~vessel geometry shape~~ the arterial shape of the aortic arch influence overall flow characteristics, including secondary flow formation and recirculation. While streamlines emphasize bulk flow paths and recirculation, vector fields resolve local velocity magnitude and direction."

Comment 10: In Fig. 3 and after, numbers of color bar should be replaced with real value

rather than xe-01 for easier reading. Explain more details of the blue lines like helical flow close to wall.

Response 10: *We thank the reviewer for this comment. All figures have been revised to display color-bar legends using decimal notation with real physical values instead of scientific notation (e.g., $\times 10^{-1}$) to improve readability. In addition, the manuscript text has been expanded to clarify the physical interpretation of the blue-colored flow structures, which correspond to low-velocity, near-wall helical and secondary flow patterns induced by the curvature of the aortic arch.*

"The diabetic case (Figures 4b and 5b presents markedly more complex flow dynamics. Streamlines exhibit pronounced curvature and bifurcation-induced deflection, particularly along the inner aortic arch and near the brachiocephalic and carotid branches, reflecting the combined effects of geometry and elevated blood viscosity. Vector plots reveal steep velocity gradients and localized high-velocity regions, indicating increased shear and flow non-uniformity. In addition, persistent blue-colored streamlines observed near the vessel wall indicate low-velocity helical and secondary flow structures, which are amplified by higher viscosity and increased viscous resistance, contributing to increased residence time and disturbed near-wall shear environments."

Comment 11: In line 225, how are these suggested marginally higher viscosity and hematocrit?

Response 11: *We thank the reviewer for this comment. The statement regarding marginally higher viscosity and hematocrit has been clarified by explicitly referencing the rheological parameters in Table 3 and the viscosity–shear-rate trends shown in Fig. 3. The revised text now explains that Healthy Case 1 exhibits a slightly higher effective viscosity than Case 2 over physiologically relevant shear rates, which accounts for the observed differences in outlet flow behavior.*

"~~Case 1 shows slightly more flow acceleration near outlets, suggesting marginally higher viscosity or hematocrit compared to Case 2.~~ Consistent with the rheological parameters in Table 3 and the viscosity–shear-rate relationship shown in Fig. 3, Healthy Case 1 exhibits a slightly higher effective viscosity than Case 2 over physiologically relevant shear rates, which manifests as modest differences in outlet velocity distributions."

Comment 12: Fig.6 & 10, the descriptions are not consistent with the bars in several expressions so please check them and rewrite them. How did you divide to three groups, red, greed, and yellow? Specify the references for each group.

Response 12: *We thank the reviewer for this comment. The descriptions associated with Figures 6 and 10 have been carefully revised to ensure consistency with the quantitative trends shown by the bar plots, avoiding overgeneralized statements and emphasizing outlet- and metric-specific variations. In addition, the grouping indicated by red, yellow, and green has been clarified as a relative, illustrative hemodynamic severity classification rather than a clinical risk metric. The basis for these groups is now explicitly described in the text, with anemic, diabetic, and healthy cases defined according to their hematocrit and viscosity levels, supported by the referenced rhe-*

ological data [1, 3]. These revisions improve clarity and alignment between the figures and their interpretations.

"Figure 6 quantifies the maximum, minimum, and average outlet velocities across all four blood conditions. ~~Diabetic blood shows consistently elevated peak and mean velocities, particularly at O2, likely due to greater cardiac effort needed to overcome increased viscosity.~~ Diabetic blood generally exhibits higher maximum and average velocities at selected outlets, particularly O2, reflecting the increased pressure gradients required to overcome elevated viscosity. ~~Anemic flow exhibits the lowest velocities, reflecting reduced hematocrit and diminished transport capacity.~~ Anemic blood tends to show lower velocity magnitudes across most outlets, consistent with reduced hematocrit and lower viscous resistance. Healthy cases cluster between these extremes, ~~with Case 1 tending toward slightly higher values.~~ with modest differences between Case 1 and Case 2 attributable to inter-individual rheological variability."

"Minimum velocities show stronger contrasts. ~~In diabetic and healthy flows, O3 and O4 demonstrate significant drops in minimum velocity, suggesting transient recirculation or localized flow deceleration—both clinically relevant~~ Reduced minimum velocities at O3 and O4 are observed for diabetic and healthy cases, indicating localized flow deceleration and intermittent recirculation, which are clinically relevant for shear-induced platelet activation and potential thrombus formation [12, 15]. ~~In contrast, the anemic profile remains relatively flat across all outlets, reflecting uniform forward flow with minimal resistance.~~ In contrast, the anemic case exhibits comparatively uniform minimum velocities across outlets, reflecting more consistent forward flow with minimal resistance."

"Figure 10b provides a ~~categorical risk assessment~~ categorical hemodynamic risk assessment intended as an illustrative tool based on minimum, maximum, and average wall pressure metrics. The classification logic is physiologically informed and intended to capture deviations from optimal hemodynamic conditions in a relative, comparative sense rather than as a clinically validated predictor. In pulsatile aortic blood flow, the minimum and maximum luminal pressures could be interpreted as approximations of diastolic and systolic pressures, respectively. Although CFD simulations typically do not model arterial wall compliance unless coupled with structural mechanics, these pressure extrema still offer useful ~~proxies for clinical pressure phases~~ hemodynamic proxies for comparative analysis when performing comparative risk analysis. The classification matrix emphasizes key ~~clinical contrasts~~ hemodynamic contrasts across blood rheological states. The diabetic profile is flagged for ~~high-pressure risk~~ elevated hemodynamic pressure severity, due to its elevated maximum wall pressure, consistent with increased vascular resistance and strain on the arterial wall. It also shows a borderline response in minimum and average pressure, suggesting incomplete normalization even during diastolic phases [11]. The anemic condition is marked by ~~low-pressure risk~~ reduced pressure severity, particularly in the minimum metric, which reflects reduced circulatory force and potential for inadequate capillary perfusion. Both healthy cases, particularly Healthy 2 fall predominantly in the ~~no-risk zone~~ low-severity zone, reinforcing the interpretation that these conditions exhibit stable, well-regulated hemodynamics supportive of systemic vascular health."

Comment 13: In line 262, why did you set 0 Pa for the outlet pressure and this doesn't seem to be physiological.

Response 13: *We appreciate the reviewer's observation. In the revised model, the constant outlet pressure assumption (0 Pa gauge) has been replaced with a physiologically realistic three-element Windkessel (RCR) model at each of the four outlets. This approach allows outlet pressure to evolve dynamically according to the instantaneous flow rate, thereby reproducing physiologic pressure–flow coupling and wave reflections. The aortic arch comprises four outlets—the brachiocephalic (O_1), left common carotid (O_2), left subclavian (O_3), and descending aorta (O_4)—each coupled to its own set of RCR parameters $(R_{1,i}, R_{2,i}, C_i)$ representing proximal resistance, distal resistance, and compliance [4, 6, 10]. Under these pulsatile inlet and RCR outlet conditions, the simulated pressure field exhibits physiologically realistic wave propagation and damping across the arch, eliminating the limitation of a fixed, non-physiological outlet pressure noted in the original submission. Accordingly, all pressure- and flow-related figures, tables, and associated discussions have been updated to reflect the revised RCR outlet boundary conditions.*

"The aortic arch model comprises four outlets: the brachiocephalic artery (O_1), left common carotid artery (O_2), left subclavian artery (O_3), and descending aorta (O_4). Each outlet was coupled with a three-element Windkessel (RCR) model to represent downstream vascular resistance and compliance [4, 6, 10]. This model provides a physiologically realistic pressure–flow coupling by accounting for both the resistive and compliant effects of the peripheral vasculature, allowing outlet pressure to evolve dynamically with time rather than remain fixed. Each branch was assigned parameters $(R_{1,i}, R_{2,i}, C_i)$, denoting proximal resistance, distal resistance, and compliance, respectively, and related through the Windkessel formulation $P_i(t) = P_{c,i}(t) + R_{1,i}Q_i(t)$, where $P_{c,i}$ varies with capacitive storage and distal resistance. The governing relation was discretized using a first-order backward-Euler scheme to ensure numerical stability:

$$P_i^{n+1} = \frac{\beta_i P_i^n + Q_i^{n+1}(R_{2,i} + R_{1,i} + R_{1,i}\beta_i) - R_{1,i}\beta_i Q_i^n}{1 + \beta_i}, \qquad \beta_i = \frac{R_{2,i}C_i}{\Delta t}. \tag{6}$$

The updated pressure P_i^{n+1} was applied at each time step as the outlet boundary condition, allowing physiological wave reflections and pressure decay throughout the cardiac cycle. The total resistance for each branch was estimated as $R_{\text{tot},i} = \bar{P}_{\text{mean}}/\bar{Q}_{i,\text{mean}}$, with $R_{1,i} = 0.05\,R_{\text{tot},i}$, $R_{2,i} = 0.95\,R_{\text{tot},i}$, and $C_i = \tau/R_{2,i}$, where $\tau \approx 1.0\,\text{s}$ is the diastolic decay constant. The mean flow partitions among the four outlets followed the ratios reported by Reymond *et al.* [10]."

"Pressure distribution in the aortic arch provides critical insights into the flow resistance and hemodynamic forces acting on the arterial walls. Under pulsatile inlet conditions with RCR outlet coupling, the simulated pressure field reflects physiologically realistic wave propagation and damping across the arch. Abnormal pressure gradients are clinically associated with increased afterload, impaired organ perfusion, and progressive cardiovascular disease, particularly in patients with hypertension or systemic vascular stiffening [11]."

Comment 14: In 347&8, how would you justify this with low hematocrit and viscosity? Can you evaluate each of these effects from hematocrit and viscosity?

Response 14: *We thank the reviewer for this comment. The manuscript has been revised to explicitly distinguish the individual roles of viscosity and hematocrit in determining the wall shear stress distribution. Reduced blood viscosity directly lowers wall shear stress through the viscous stress term in the momentum balance, while low hematocrit contributes indirectly by smoothing velocity profiles and reducing near-wall shear gradients. This clarification has been added to the revised text to improve the physical justification of the observed WSS patterns in the anemic case.*

"Figure 11 shows the spatial distribution of WSS under four blood conditions. The anemic case exhibits smooth, uniform flow with moderate WSS magnitudes throughout the aortic arch. ~~This is attributed to low hematocrit and viscosity, which reduce flow resistance and minimize sharp velocity gradients.~~ This behavior can be attributed to reduced blood viscosity, which directly lowers wall shear stress for a given velocity gradient, and to low hematocrit, which indirectly contributes by smoothing velocity profiles and reducing near-wall shear gradients."

—

Before closing, we would like to express our sincere thanks for your kind advice and careful review, which have greatly helped us improve our paper. We hope our revisions and explanations meet your expectations and suggestions. Thank you very much!

References

[1] H. Ahmed and C. Podder. Hemodynamical behavior analysis of anemic, diabetic, and healthy blood flow in the carotid artery. *Heliyon*, 10(4):e26622, Feb. 2024. ISSN 2405-8440. doi: 10.1016/j.heliyon.2024.e26622.

[2] I. Akinsheye and E. S. Klings. Sickle cell anemia and vascular dysfunction: The nitric oxide connection. *Journal of Cellular Physiology*, 224(3):620–625, June 2010. ISSN 1097-4652. doi: 10.1002/jcp.22195.

[3] R. M. Attia and M. A. A. Eldosoky. Studying of the blood flow behavior in a stenosed carotid artery for healthy, anemic and diabetic blood. In *2020 International Conference on Innovative Trends in Communication and Computer Engineering (ITCE)*, page 72–75. IEEE, Feb. 2020. doi: 10.1109/itce48509.2020.9047796.

[4] S. M. Black, C. Maclean, P. Hall Barrientos, K. Ritos, A. McQueen, and A. Kazakidi. Calibration of patient-specific boundary conditions for coupled cfd models of the aorta derived from 4d flow-mri. *Frontiers in Bioengineering and Biotechnology*, 11, May 2023. ISSN 2296-4185. doi: 10.3389/fbioe.2023.1178483.

[5] S. L. Diamond. Systems analysis of thrombus formation. *Circulation Research*, 118(9): 1348–1362, Apr. 2016. ISSN 1524-4571. doi: 10.1161/circresaha.115.306824.

[6] H. Gharahi, B. A. Zambrano, D. C. Zhu, J. K. DeMarco, and S. Baek. Computational fluid dynamic simulation of human carotid artery bifurcation based on anatomy and volumetric blood flow rate measured with magnetic resonance imaging. *International Journal of Advances in Engineering Sciences and Applied Mathematics*, 8(1):46–60, Feb. 2016. ISSN 0975-5616. doi: 10.1007/s12572-016-0161-6.

[7] K. Giannokostas and Y. Dimakopoulos. Tevp model predictions of the pulsatile blood flow in 3d aneurysmal geometries. *Journal of Non-Newtonian Fluid Mechanics*, 311:104969, 2023. ISSN 0377-0257. doi: 10.1016/j.jnnfm.2022.104969.

[8] K. Giannokostas, Y. Dimakopoulos, A. Anayiotos, and J. Tsamopoulos. Advanced constitutive modeling of the thixotropic elasto-visco-plastic behavior of blood: Steady-state blood flow in microtubes. *Materials*, 14(2):367, 2021. ISSN 1996-1944. doi: 10.3390/ma14020367.

[9] A. M. Malek. Hemodynamic shear stress and its role in atherosclerosis. *JAMA*, 282 (21):2035, Dec. 1999. ISSN 0098-7484. doi: 10.1001/jama.282.21.2035.

[10] P. Reymond, F. Merenda, F. Perren, D. Rüfenacht, and N. Stergiopulos. Validation of a one-dimensional model of the systemic arterial tree. *American Journal of Physiology-Heart and Circulatory Physiology*, 297(1):H208–H222, July 2009. ISSN 1522-1539. doi: 10.1152/ajpheart.00037.2009.

[11] M. E. Safar. Arterial stiffness as a risk factor for clinical hypertension. *Nature Reviews Cardiology*, 15(2):97–105, Oct. 2017. ISSN 1759-5010. doi: 10.1038/nrcardio.2017.155.

[12] D. Steinman, D. Vorp, and C. Ethier. Computational modeling of arterial biomechanics: Insights into pathogenesis and treatment of vascular disease. *Journal of Vascular Surgery*, 37(5):1118–1128, May 2003. ISSN 0741-5214. doi: 10.1067/mva.2003.122.

[13] P. H. Stone, S. Saito, and et al. Prediction of progression of coronary artery disease and clinical outcomes using vascular profiling of endothelial shear stress and arterial plaque characteristics: The prediction study. *Circulation*, 126(2):172–181, July 2012. ISSN 1524-4539. doi: 10.1161/circulationaha.112.096438.

[14] Z. Sun and L. Xu. Computational fluid dynamics in coronary artery disease. *Computerized Medical Imaging and Graphics*, 38(8):651–663, Dec. 2014. ISSN 0895-6111. doi: 10.1016/j.compmedimag.2014.09.002.

[15] L. Xu, T. Yang, L. Yin, Y. Kong, Y. Vassilevski, and F. Liang. Numerical simulation of blood flow in aorta with dilation: A comparison between laminar and les modeling methods. *Computer Modeling in Engineering & Sciences*, 124(2):509–526, 2020. ISSN 1526-1506. doi: 10.32604/cmes.2020.010719.

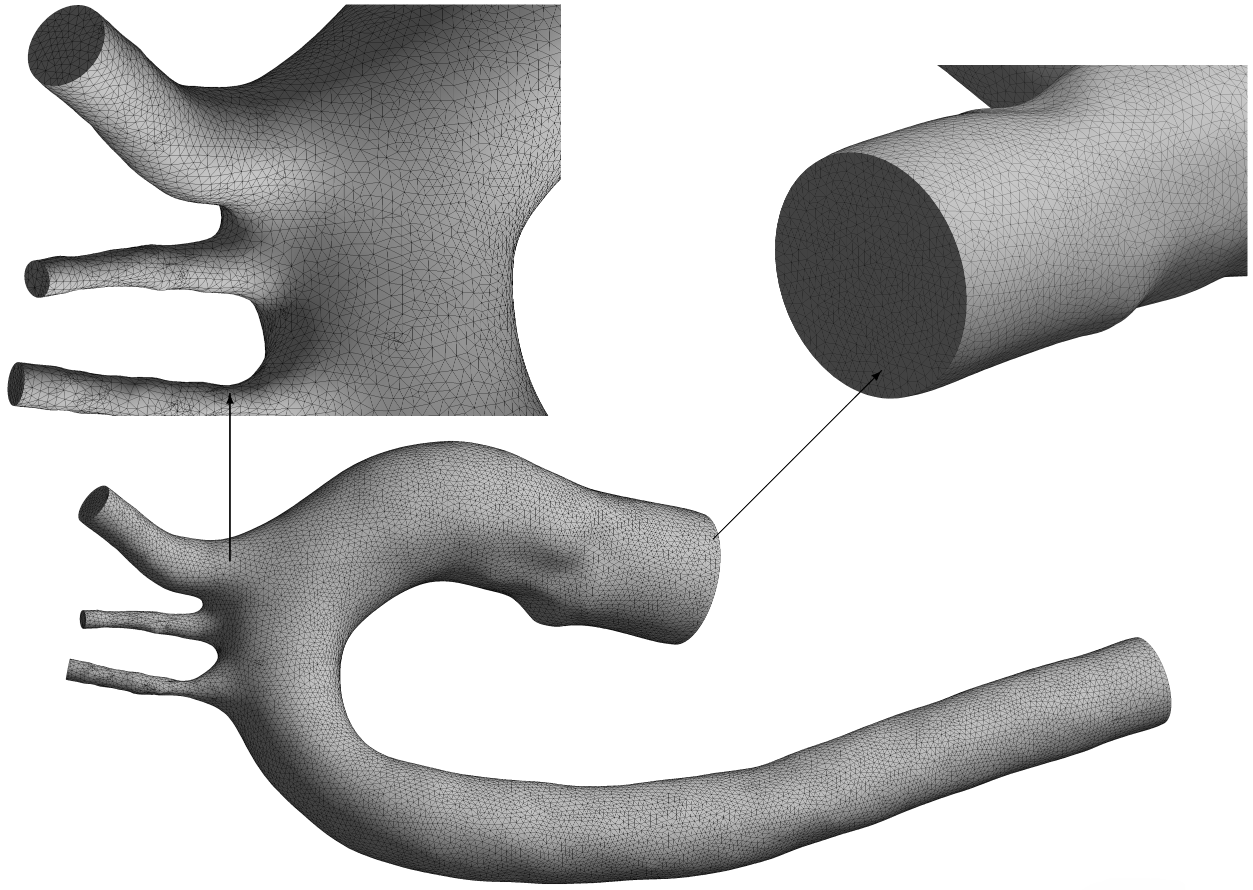

Figure 2: **Unstructured tetrahedral mesh of the aortic arch. Enlarged views on the left highlight local mesh refinement at a supra-aortic branch junction, while the view on the right illustrates near-wall resolution at the inlet.**

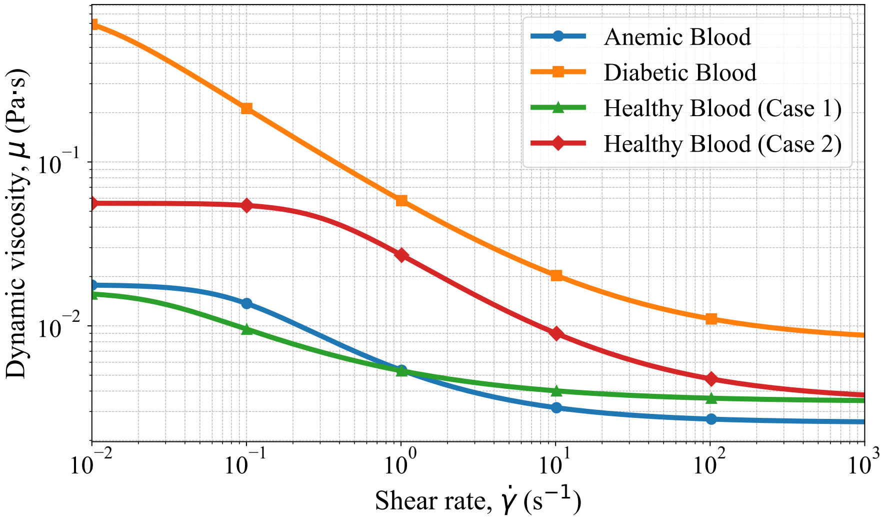

Figure 3: **Variation of effective blood viscosity with shear rate for four blood conditions based on the Carreau–Yasuda model defined in Eq. (5).**

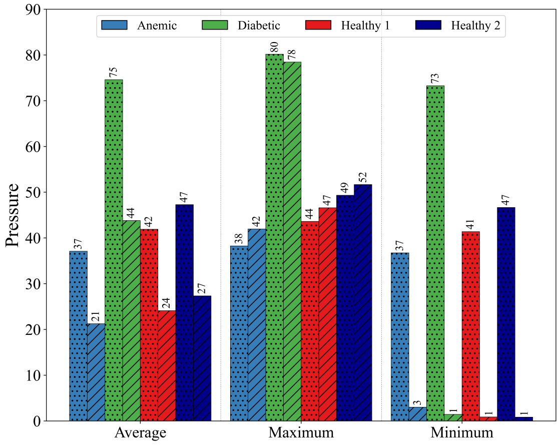

(a) Pressure distribution

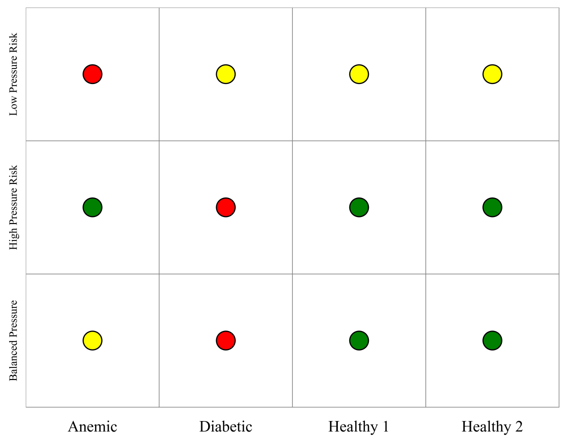

(b) Pressure-based risk classification.

Figure 10: **(a) Pressure distribution across four blood conditions: anemic, diabetic, healthy (Case 1), and healthy (Case 2) showing average, maximum, and minimum values. Dotted bars indicate inlet pressure, while diagonally hatched bars represent wall pressure. Diabetic cases exhibit elevated pressure magnitudes at both inlet and wall locations, while anemic cases show considerably lower wall pressure values. (b) Pressure-based ~~risk classification~~ hemodynamic risk classification (illustrative) using three metrics: minimum, maximum, and average pressure. Color-coded circles represent ~~risk levels~~ relative hemodynamic severity levels, where red indicates high, yellow indicates borderline or moderate concern, and green indicates no risk.**

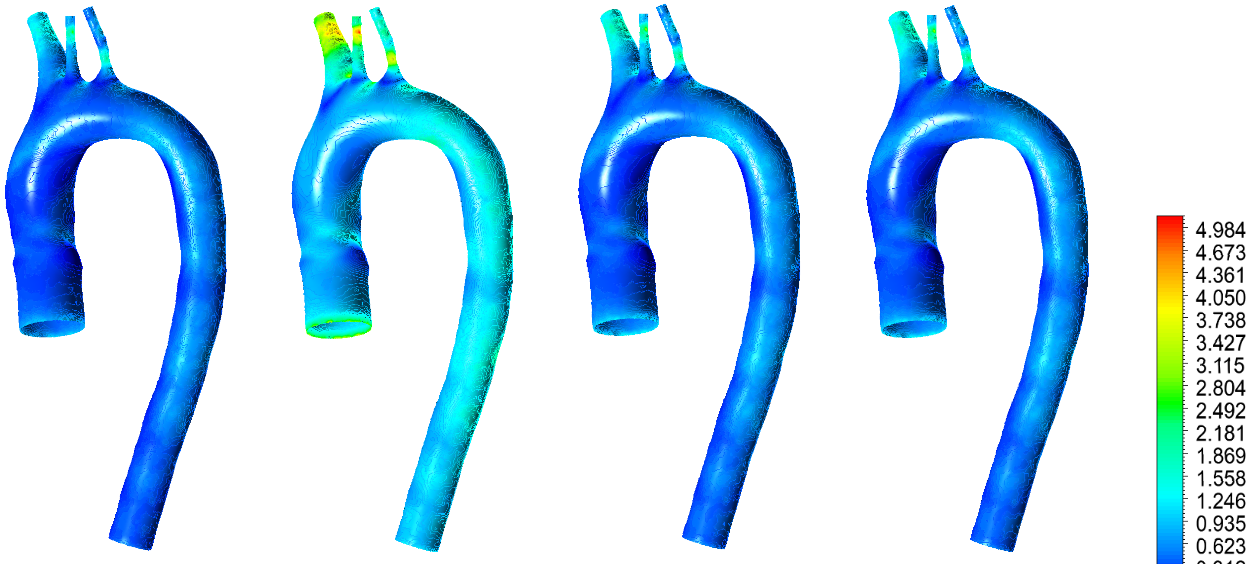

(a) Anemic (b) Diabetic (c) Healthy 1 (d) Healthy 2

Figure 11: **Wall shear stress (WSS) contours in the aortic arch for different blood flow cases: (a) anemic, (b) diabetic, (c) healthy (Case 1), and (d) healthy (Case 2). Red regions correspond to higher WSS values, while blue regions indicate lower WSS values in Pascal (Pa).**

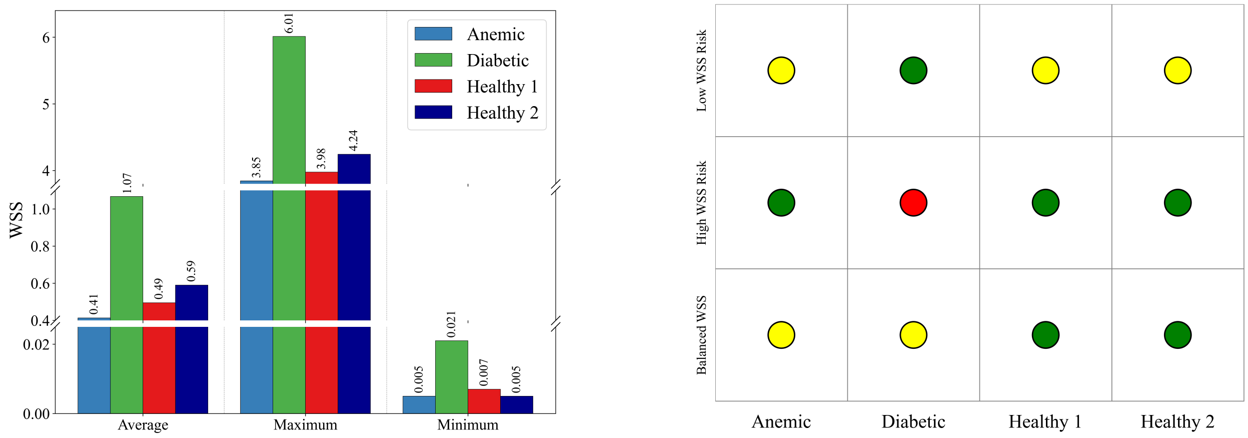

(a) WSS distribution (b) WSS-based risk classification

Figure 12: **(a) WSS distribution across four blood conditions: anemic, diabetic, healthy (Case 1), and healthy (Case 2) showing average, maximum, and minimum values. A broken y-axis is used to accommodate both low (below 0.05 Pa) and high (above 1 Pa) WSS values. (b) ~~Risk classification~~ Illustrative hemodynamic risk classification based on three WSS metrics: minimum, maximum, and average WSS. Each cell is color-coded to represent the ~~severity of the condition~~ relative hemodynamic severity under a given metric, where red indicates high, yellow indicates borderline or moderate concern, and green indicates no risk.**
