# Supplementary material for: Hemodynamic simulation in the aortic arch under anemic, diabetic, and healthy blood flow conditions using computational fluid dynamics
Source: PLoS One. 2026 Mar 11;21(3):e0334297. doi: 10.1371/journal.pone.0334297 (PMC12978756; doi:10.1371/journal.pone.0334297)
Supplement: Attachment — Submitted filename: Response to reviewers2.pdf [file pone.0334297.s004.pdf]

For research article

Response to Reviewer Comments

Response to Reviewer 1

All comments have been addressed. I propose the acceptance of the manuscript for publishing. The authors have addressed all issues mentioned in my previous report.

Response: *We sincerely thank the reviewer for the careful evaluation of our manuscript and for acknowledging that the revisions have satisfactorily addressed all previously raised concerns. We truly appreciate your constructive feedback throughout the review process, which has helped improve the clarity and quality of the work.*

Response to Reviewer 2

We sincerely thank the reviewer for the careful re-evaluation of our revised manuscript and for the constructive and insightful comments. We greatly appreciate the reviewer's observations regarding shear-dependent viscosity interpretation and its hemodynamic implications. In response, we have provided quantitative comparisons across representative shear ranges, explicitly distinguished between low-shear aggregation-dominated behavior and the moderate-to-high shear regime characteristic of large-artery flow, standardized the color scheme across all figures for clarity, and expanded the limitations section to acknowledge spatiotemporal red blood cell aggregation and hematocrit redistribution effects. These revisions have strengthened the clarity, rigor, and overall quality of the manuscript, and we are grateful for the reviewer's valuable feedback.

Most of the review comments were satisfactorily answered. However, there are some more which should be addressed.

Response: *We sincerely thank the reviewer for the careful re-evaluation of our revised manuscript and for acknowledging that most of the concerns have been satisfactorily addressed. We appreciate the opportunity to further improve the manuscript and have carefully addressed the additional comments below.*

Comment 1: There are little differences of dynamic viscosity with shear rate between anemic blood and healthy blood 1. Rather there are more differences between two healthy bloods. Therefore, the results would be following those differences by the CFD. Please quantify the differences between 2 healthy subjects and anemic and healthy blood 1. Add the discussion this point too.

Response 1: *We thank the reviewer for this important observation. To address this comment, we have quantified the relative differences in dynamic viscosity between Healthy Subject 1 and*

Healthy Subject 2, and between the anemic case and Healthy Subject 1, across representative shear rates.

At very low shear rates (0.01–1 s^{-1}), the viscosity difference between the two healthy subjects is indeed larger than the difference between the anemic case and Healthy Subject 1. For example, at 0.01 s^{-1}, Healthy Subject 2 exhibits approximately three to four times higher viscosity than Healthy Subject 1, whereas the anemic case differs from Healthy Subject 1 by less than 20%. This confirms that inter-healthy variability is more pronounced in the aggregation-dominated low-shear regime.

However, in the physiologically relevant arterial shear range (10–100 s^{-1}), which characterizes most of the cardiac cycle in the aortic arch, the viscosity differences become smaller and of comparable magnitude. At 100 s^{-1}, Healthy Subject 2 is approximately 25–30% more viscous than Healthy Subject 1, while the anemic case is approximately 25–30% less viscous than Healthy Subject 1. Therefore, within the arterial shear regime, the deviation from Healthy Subject 1 is of similar order for both Healthy Subject 2 and the anemic case.

Consistently, the CFD results reflect this effective viscosity behavior within the arterial shear range rather than the extreme low-shear regime. For example, the maximum wall shear stress differs by approximately 6–7% between Healthy Subject 2 and Healthy Subject 1, and by approximately 3–4% between Healthy Subject 1 and the anemic case. The pressure fields exhibit the same systematic ordering across all cases.

Since large-artery hemodynamics are predominantly governed by moderate-to-high shear conditions and inertia-dominated pulsatile flow [2], the observed hemodynamic variations correspond to the shear-dependent effective viscosity within this arterial regime. The manuscript has been revised to include these quantitative comparisons and corresponding discussion for clarity.

"Figure 3 illustrates the variation of effective blood viscosity with shear rate for the four blood conditions computed using the Carreau–Yasuda formulation in Eq. (**??**). In all cases, viscosity decreases monotonically with increasing shear rate, demonstrating the characteristic shear-thinning behavior of blood. Diabetic blood exhibits the highest viscosity across the entire shear-rate range due to elevated zero-shear viscosity and a delayed transition to the high-shear plateau, whereas anemic blood shows the lowest viscosity ~~with a more rapid reduction as shear rate increases~~ in the moderate-to-high shear regime relevant to large-artery flow. The two healthy blood cases fall between these extremes, indicating moderate shear-thinning behavior and inter-individual rheological variability. Although the difference between the two healthy subjects is more pronounced in the very low-shear range (< 1 s^{-1}), the viscosity deviations from Healthy Case 1 become comparable in magnitude for both Healthy Case 2 and the anemic case within the arterial shear range ($\mathcal{O}(10^2$–$10^3)$ s^{-1}), consistent with shear rates estimated from reported mean arterial wall shear stresses (approximately 1–2 Pa) in large arteries [1, 2]. Through Eq. (3), these shear-dependent viscosity variations directly modulate the viscous stress term in the momentum equations, thereby influencing local velocity gradients and wall shear stress distributions, ~~particularly in near-wall and low-shear regions of the aortic arch~~ throughout the pulsatile arterial flow field. Based on the computed wall shear stresses and corresponding effective viscosity values, the estimated wall shear rates are on the order of several hundred s^{-1}, confirming that the present aortic flow operates predominantly within this moderate-to-high shear regime characteristic of large arteries [1, 2]."

Comment 2: Authors described that anemic blood shows the lowest viscosity, but that's true for only $> 1s^{-1}$. Low shear rate is more important for viscosity and blood flow. This should be carefully analyzed and discussed.

Response 2: *We sincerely thank the reviewer for this important and thoughtful comment. We agree that at very low shear rates ($< 1\ \mathrm{s}^{-1}$), viscosity differences between blood conditions are reduced and red blood cell aggregation effects become more dominant. In this regime, the distinction between anemic and healthy blood is indeed less pronounced compared to moderate-to-high shear rates.*

However, the present study investigates large-artery hemodynamics in the aortic arch. Based on the computed wall shear stresses (3–6 Pa) and the corresponding effective viscosity values, the estimated wall shear rates are on the order of several hundred s^{-1}. This confirms that the bulk aortic flow operates predominantly within the moderate-to-high shear regime ($\mathcal{O}(10^2–10^3)\ \mathrm{s}^{-1}$) characteristic of inertia-dominated arterial flow [2].

Within this physiologically relevant arterial shear range, anemic blood consistently exhibits lower effective viscosity than the healthy cases, while diabetic blood remains the highest across the entire shear spectrum. To clarify this distinction, we have revised the manuscript to explicitly differentiate between low-shear aggregation behavior and the moderate-to-high shear regime characteristic of large arteries.

"Figure 3 illustrates the variation of effective blood viscosity with shear rate for the four blood conditions computed using the Carreau–Yasuda formulation in Eq. (**??**). In all cases, viscosity decreases monotonically with increasing shear rate, demonstrating the characteristic shear-thinning behavior of blood. Diabetic blood exhibits the highest viscosity across the entire shear-rate range due to elevated zero-shear viscosity and a delayed transition to the high-shear plateau, whereas anemic blood shows the lowest viscosity ~~with a more rapid reduction as shear rate increases~~ in the moderate-to-high shear regime relevant to large-artery flow. The two healthy blood cases fall between these extremes, indicating moderate shear-thinning behavior and inter-individual rheological variability. Although the difference between the two healthy subjects is more pronounced in the very low-shear range ($< 1\ \mathrm{s}^{-1}$), the viscosity deviations from Healthy Case 1 become comparable in magnitude for both Healthy Case 2 and the anemic case within the arterial shear range ($\mathcal{O}(10^2–10^3)\ \mathrm{s}^{-1}$), consistent with shear rates estimated from reported mean arterial wall shear stresses (approximately 1–2 Pa) in large arteries [1, 2]. Through Eq. (3), these shear-dependent viscosity variations directly modulate the viscous stress term in the momentum equations, thereby influencing local velocity gradients and wall shear stress distributions, ~~particularly in near-wall and low-shear regions of the aortic arch~~ throughout the pulsatile arterial flow field. Based on the computed wall shear stresses and corresponding effective viscosity values, the estimated wall shear rates are on the order of several hundred s^{-1}, confirming that the present aortic flow operates predominantly within this moderate-to-high shear regime characteristic of large arteries [1, 2]."

Comment 3: The color of 4 cases should be consistent for all figures for easy readibility.

Response 3: *We thank the reviewer for this helpful suggestion. The color scheme has been standardized across all figures for consistency and improved readability. Specifically, Figures 3, 6, 10a, and 12a have been updated so that each blood condition is represented by the same color throughout the manuscript.*

Comment 4: The red blood cell aggregation and viscosity as a function of axial shear rate in addition to radial shear rate and local hematocrit under pulsatile flow are spatiotemporal variations and these are not discussed fully without any references. Even though this study could not integrate these spatiotemporal variations, it's important to be discussed as a limitation. It may be more important for pathological blood between healthy and anemic or diabetic bloods since there are more possibilities that their red cell aggregation tendency would be different, which are not fully reflected by the viscosity function as a shear rate.

Response 4: *We thank the reviewer for this insightful comment. The limitation section has been expanded to explicitly acknowledge that axial and radial shear variations, local hematocrit redistribution, and spatiotemporal red blood cell aggregation effects were not modeled within the present shear-rate–dependent Carreau framework. We have clarified that these microstructural effects may be more pronounced in pathological blood conditions and may not be fully captured by a viscosity function based solely on instantaneous shear rate. These considerations are now clearly stated as limitations and directions for future work.*

"Despite these insights, the study has certain limitations. The simulations were conducted on a generalized aortic geometry rather than patient-specific models, which may limit anatomical specificity. Blood flow was assumed to be laminar and incompressible, with rigid arterial walls and thus excluding potential contributions from fluid-structure interaction and wall elasticity. Additionally, while the Carreau model captures shear-thinning effects, it does not account for pathological viscoplasticity seen in conditions like thrombosis or inflammation. Moreover, shear-rate dependent viscosity models do not explicitly resolve time-dependent red blood cell aggregation, shear-history effects, or spatial variations in aggregation dynamics that may arise under pulsatile flow. In particular, axial and radial shear variations and local hematocrit redistribution were not modeled, and such spatiotemporal microstructural effects may be more pronounced in pathological blood where aggregation behavior differs. Experimental validation was beyond the scope of this work but remains essential to confirm key predictions through experimental (e.g., particle image velocimetry) or clinical imaging (e.g., 4D Flow MRI) studies, particularly regarding shear stress thresholds and risk classification."

—

Before closing, we would like to express our sincere thanks for your careful review, which have greatly helped us to further improve our paper. We hope our revisions and explanations meet your expectations and suggestions. Thank you very much!

References

[1] S. A. Berger and L.-D. Jou. Flows in stenotic vessels. *Annual Review of Fluid Mechanics*, 32(1):347–382, 2000. ISSN 1545-4479. doi: 10.1146/annurev.fluid.32.1.347.

[2] D. N. Ku. Blood flow in arteries. *Annual Review of Fluid Mechanics*, 29(1):399–434, 1997. ISSN 1545-4479. doi: 10.1146/annurev.fluid.29.1.399.

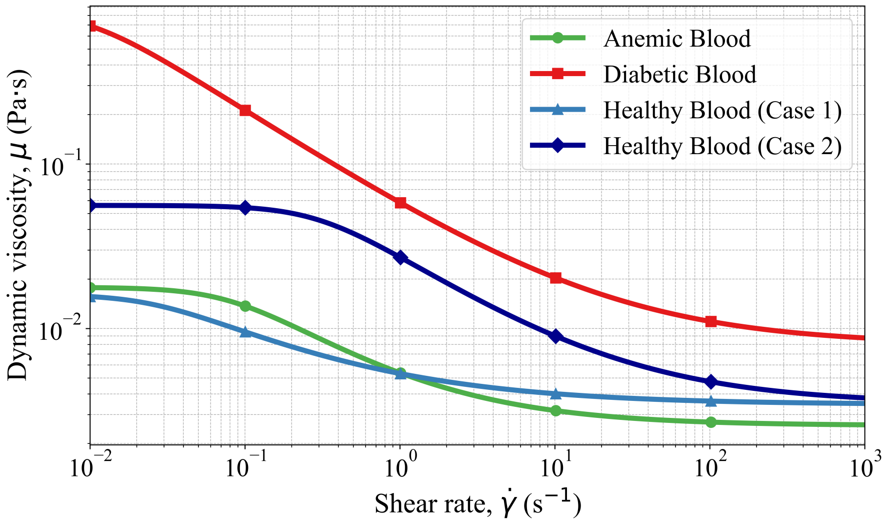

Figure 3: **Variation of effective blood viscosity with shear rate for four blood conditions based on the Carreau–Yasuda model defined in Eq. 3.**

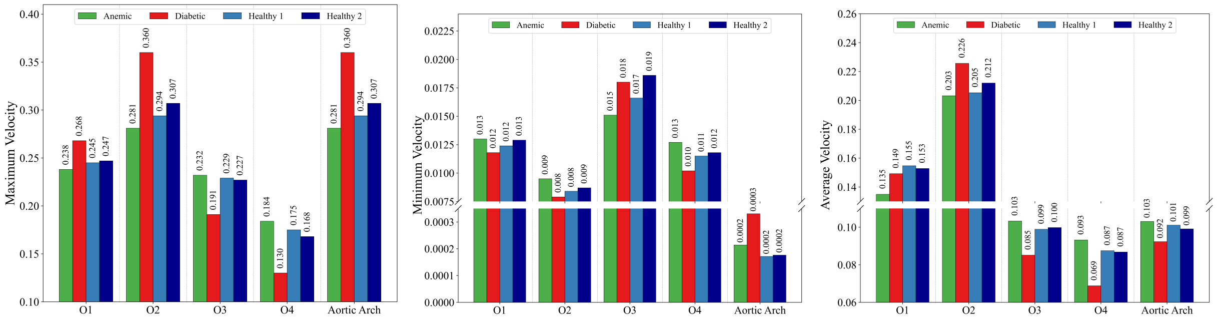

Figure 6: **Velocity distributions: average (left), minimum (middle), and maximum (right) across different outlets and cases.**

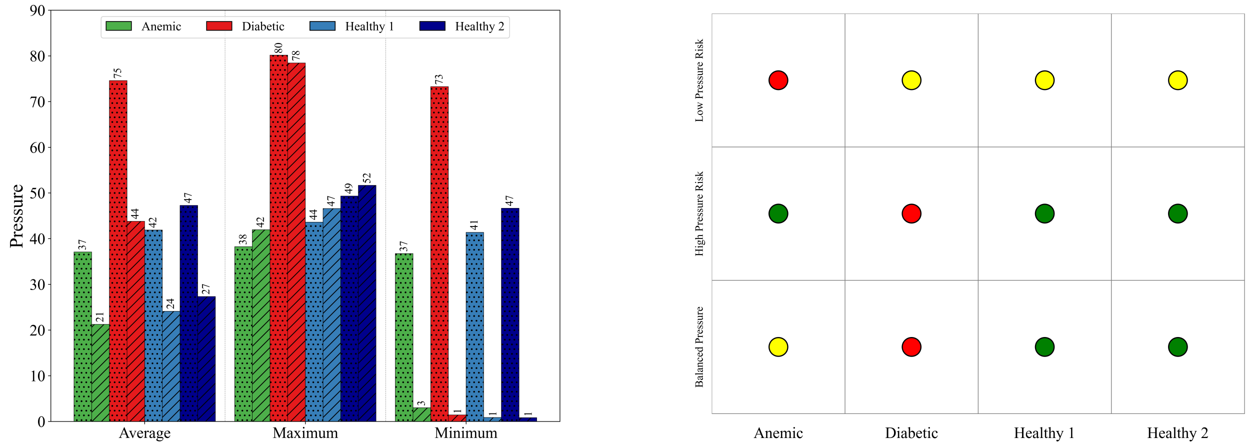

(a) Pressure distribution

(b) Pressure-based risk classification.

Figure 10: **(a) Pressure distribution across four blood conditions: anemic, diabetic, healthy (Case 1), and healthy (Case 2) showing average, maximum, and minimum values. Dotted bars indicate inlet pressure, while diagonally hatched bars represent wall pressure. Diabetic cases exhibit elevated pressure magnitudes at both inlet and wall locations, while anemic cases show comparatively lower wall pressure values. (b) Pressure-based hemodynamic risk classification (illustrative) using three metrics: minimum, maximum, and average pressure. Color-coded circles represent relative hemodynamic severity levels, where red indicates high, yellow indicates borderline or moderate concern, and green indicates no risk.**

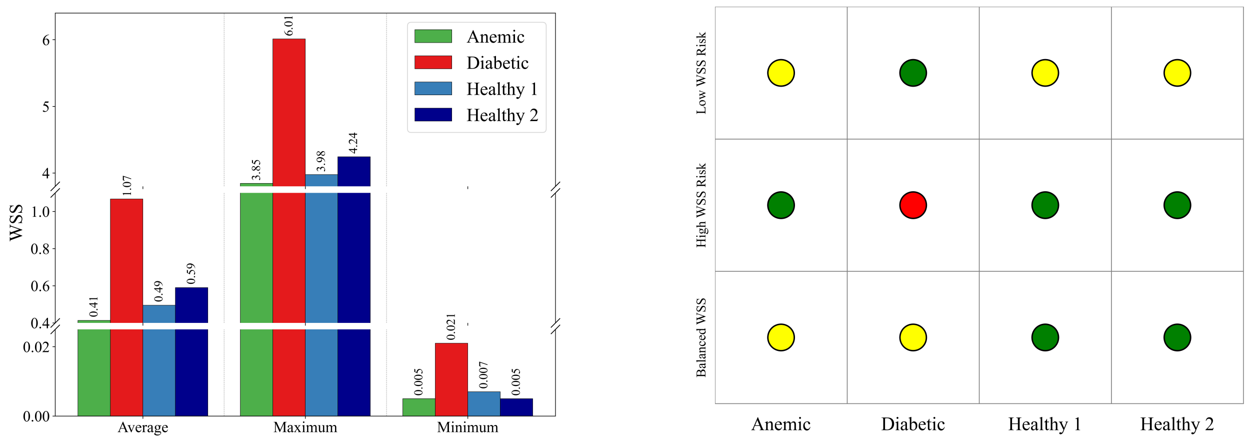

(a) WSS distribution

(b) WSS-based risk classification

Figure 12: **(a) WSS distribution across four blood conditions–anemic, diabetic, healthy (Case 1), and healthy (Case 2)–showing average, maximum, and minimum values. A broken y-axis is used to accommodate both low (below 0.05 Pa) and high (above 1 Pa) WSS values. (b) Illustrative hemodynamic risk classification based on three WSS metrics: minimum, maximum, and average WSS. Each cell is color-coded to represent the relative hemodynamic severity under a given metric, where red indicates high, yellow indicates borderline or moderate concern, and green indicates no risk.**
