# Supplementary material for: Hemodynamic simulation in the aortic arch under anemic, diabetic, and healthy blood flow conditions using computational fluid dynamics
Source: PLoS One. 2026 Mar 11;21(3):e0334297. doi: 10.1371/journal.pone.0334297 (PMC12978756; doi:10.1371/journal.pone.0334297)
Supplement: Attachment — Submitted filename: PONE-D-25-52209 Review comments.docx [file pone.0334297.s001.docx]

This paper simulated the flow based on CFD in the human aortic arch for people, anemic, diabetic, and 2 healthy subjects with Carreau-Yasuda model of hemorheology under pulsatile flow. Even though this study combines hemodynamics and hemorheology with quantitative analysis, this requires extensive revision before publication.

1. Compared with previous published papers such as [7] & [20] in the reference, what is the originality of this paper? The authors should persuade this more rigorously.
2. The authors claimed that this CFD study is a direct comparison of pathological and physiological blood states for rheological interpretation under identical boundary and geometric conditions. However, it’s not clear how pathological blood would be reflected in the rheological effects in CFD. If authors meant that Carreau viscosity model with different coefficients according to pathology, are they good enough? Especially under pulsatile flow, red blood cell aggregation is not just a function of shear rate only but also a function of time and radial and axial positions depending on acceleration and hematocrit. Red cell aggregations are also dependent on axial shear rate in addition to radial shear rate under pulsatile flow. How these complicated red cell aggregation phenomena would be reflected in this simple model? Please state this with references.
3. Discussion needs to be separated from the results, and limitations need to be part of discussion rather than conclusion.
4. For governing equations, please clarify whether they are in cylindrical coordinate or cartesian coordinate including helicity eq. (5), For Eqs (3) & (4), n are not the same so different notation is required.
5. What kind of medical imaging data was used? More specific description is required in line 130.
6. In Fig. 2, (a) is not required, but (b) with more specific mesh and grid should be included in addition to overall mesh map.
7. Table 1 & 2, it not clear why 1mm of grid size and 0.4 s are optimal.
8. In addition to Table 3, the graphs are required to figure it out how viscosity would be changed with shear rate for 4 different blood samples.
9. In line 197 & 198, vessel geometry 197 shape overall flow characteristics should be rewritten.
10. In Fig. 3 and after, numbers of color bar should be replaced with real value rather than xe-01 for easier reading. Explain more details of the blue lines like helical flow close to wall.
11. In line 225, how are these suggested marginally higher viscosity and hematocrit?
12. Fig.5 & 9, the descriptions are not consistent with the bars in several expressions so please check them and rewrite them. How did you divide to three groups, red, greed, and yellow? Specify the references for each group.
13. In line 262, why did you set 0 Pa for the outlet pressure and this doesn’t seem to be physiological.
14. In 347&8, how would you justify this with low hematocrit and viscosity? Can you evaluate each of these effects from hematocrit and viscosity?
