# Supplementary material for: Hemodynamic simulation in the aortic arch under anemic, diabetic, and healthy blood flow conditions using computational fluid dynamics
Source: PLoS One. 2026 Mar 11;21(3):e0334297. doi: 10.1371/journal.pone.0334297 (PMC12978756; doi:10.1371/journal.pone.0334297)
Supplement: Attachment — Submitted filename: PONE-D-25-52209-2ndComments.docx [file pone.0334297.s003.docx]

Most of the review comments were satisfactorily answered. However, there are some more which should be addressed.

1. There are little differences of dynamic viscosity with shear rate between anemic blood and healthy blood 1. Rather there are more differences between two healthy bloods. Therefore, the results would be following those differences by the CFD. Please quantify the differences between 2 healthy subjects and anemic and healthy blood 1. Add the discussion this point too.
2. Authors described that anemic blood shows the lowest viscosity, but that’s true for only > 1 s^-1^. Low shear rate is more important for viscosity and blood flow. This should be carefully analyzed and discussed.
3. The color of 4 cases should be consistent for all figures for easy readibility.
4. The red blood cell aggregation and viscosity as a function of axial shear rate in addition to radial shear rate and local hematocrit under pulsatile flow are spatiotemporal variations and these are not discussed fully without any references. Even though this study could not integrate these spatiotemporal variations, it’s important to be discussed as a limitation. It may be more important for pathological blood between healthy and anemic or diabetic bloods since there are more possibilities that their red cell aggregation tendency would be different, which are not fully reflected by the viscosity function as a shear rate.
